# Distribution and curing reactions of melamine formaldehyde resin in cells of impregnation-modified wood

**DOI:** 10.1038/s41598-020-60418-3

**Published:** 2020-02-25

**Authors:** Michael Altgen, Muhammad Awais, Daniela Altgen, André Klüppel, Mikko Mäkelä, Lauri Rautkari

**Affiliations:** 1 Aalto University, School of Chemical Engineering, Department of Bioproducts and Biosystems, P.O. Box 16300, 00076 Aalto, Finland; 2 Georg-August University of Göttingen, Faculty of Forest Science and Forest Ecology, Wood Biology and Wood Products, Büsgenweg 4, 37077 Göttingen, Germany; 3 Swedish University of Agricultural Sciences, Department of Forest Biomaterials and Technology, Skogsmarksgränd, 90183 Umeå Sweden

**Keywords:** Biomaterials - cells, Composites, Imaging techniques, Microscopy

## Abstract

Wood modification improves the properties of wood as a building material by altering the wood structure on a cellular level. This study investigated how dimensional changes of wood on a macroscopic scale are related to the cellular level chemical changes on the micron level after impregnation modification with melamine formaldehyde (MF) resin under different heat curing conditions. Our results showed that the curing conditions affected the polycondensation reactions and the morphological structure of the MF resin within the cell lumen. The diffusion of the resin into the cell wall was estimated based on the triazine ring vibration of melamine in the Raman spectrum at 950–990 cm^−1^. Thereby, it was shown that macroscopic changes in wood dimensions do not provide a reliable estimate for the cell wall diffusion of the resin. The removal of cell wall constituents during the modification, which was facilitated by the alkaline pH of the impregnation solution, counterbalanced the cell wall bulking effect of the resin. This was particularly evident for wet cured samples, where diffusion of MF resin into the cell wall was observed by confocal Raman microscopy, despite a reduction in macroscopic wood dimensions.

## Introduction

Wood is a hierarchical structured biocomposite with cell walls that are built up of stiff cellulose fibrils embedded in an amorphous matrix of hemicelluloses and lignin. The main structural elements in softwood species are tracheids, which are hollow cells that are about 2–4 mm in length, 20–50 µm in diameter and have a cell wall thickness of about 2–10 µm^1,2^. This structure provides wood with a high strength to weight ratio^3^, but the hygroscopic behaviour of wood is associated with dimensional changes^4^ and degradation by decay fungi^5^. These moisture-related problems can be reduced significantly by impregnation modification using thermosetting resins, such as melamine formaldehyde (MF) resin. However, improving the dimensional stability and decay resistance of wood requires the insertion of the resin into the cell wall microstructure and cannot be realized by only filling the cell lumen^6–8^. Despite the importance of the location of the resin within the hierarchical structure of wood, our current understanding of the structure-property relationship of modified wood is mostly derived from analyses on a macroscopic scale. Only few studies have combined measurements of macroscopic wood properties with cellular level analyses of the modification agent on the micron level^9,10^.

MF resins are formed by reaction of the primary amino groups of melamine with formaldehyde to form methylol melamines with up to six methylol groups. Methylol melamines react to macromolecules by forming methylene bridges or ether bonds through self-condensation^11^. The methylol melamine can be methylated using methanol to limit self-condensation during storage and to improve miscibility with water^12^. The curing reactions and the resulting structure of the melamine resin depend on a number of factors, including the molar ratios of the reactants, the temperature of the reaction and the pH^13–15^. The cure of MF resin by heat is accelerated in the presence of wood^16,17^, but it remains unknown if this is accompanied by a change in the MF resin structure compared to the cure of pure MF resin.

**Figure 1:**
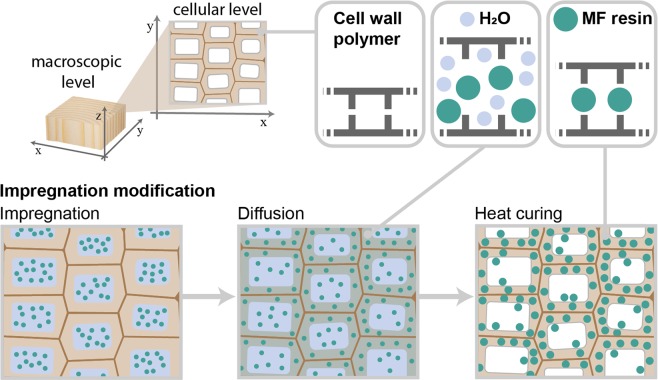
Schematic illustration of the different process steps during impregnation modification with MF resin to cause a permanent cell wall bulking effect by occupying space between wood cell wall polymers.

Low molecular weight MF and other thermosetting resins are capable of penetrating the cell wall and can be used for impregnation modification using water as a solvent^8,18,19^. The modification process requires only few process steps (see Fig. 1), starting with the impregnation of the wood with an aqueous monomer or oligomer solution using vacuum and/or pressure. Thereby, the pressure gradients generate a flow of the solution into the coarse wood structure (i.e. the cell lumens). The solvent swells the wood and, thus, enables the diffusion of the solute into the cell wall along the concentration gradient. Finally, heat curing induces the polymerization of the thermosetting resins to macromolecules. The cured thermosetting resin is irreversibly fixed within the cell walls, where it fills free space between the wood polymers to keep the wood in a permanently swollen state. This effect is commonly denoted as “cell wall bulking”. Thereby, the uptake of liquid water and water vapour into the cell wall is reduced, which limits the dimensional changes of wood^18^ and enhances its decay resistance^20^. This cell wall bulking effect is commonly determined at a macroscopic level by the increase in dry sample dimensions.

There is a number of factors that significantly affect the diffusion of resins into the wood cell walls during impregnation modification^21–23^. One major factor may be the wood moisture content during heat curing. By keeping the impregnated wood in wet state while increasing the temperature gradually during the curing (“wet curing”) instead of curing the wood in dry state (“dry curing”), a cell wall bulking effect can no longer be measured through changes in the wood sample dimensions^23^. Previously, this was assigned to a reduced cell wall diffusion during wet curing. During wet curing the impregnated samples are heated to elevated temperatures before wood drying occurs. Thereby, thermosetting resins are believed to form macromolecules that are too large to enter the cell wall pores before the diffusion is facilitated by the removal of the solvent from the lumen^23^.

Although the diffusion of MF and other thermosetting resins into wood cell walls has already been evidenced^19,24,25^, our understanding of the effect of different modification conditions on the cell wall diffusion is still lacking and is mostly derived from analyses on a macroscopic level. Our present study combined the determination of macroscopic changes of wood caused by the modification with MF resin under different curing conditions with cellular level analyses of the curing reactions and the distribution of the MF resin using scanning electron and confocal Raman microscopy.

## Results and Discussion

### Changes in sample mass and dimensions

**Figure 2:**
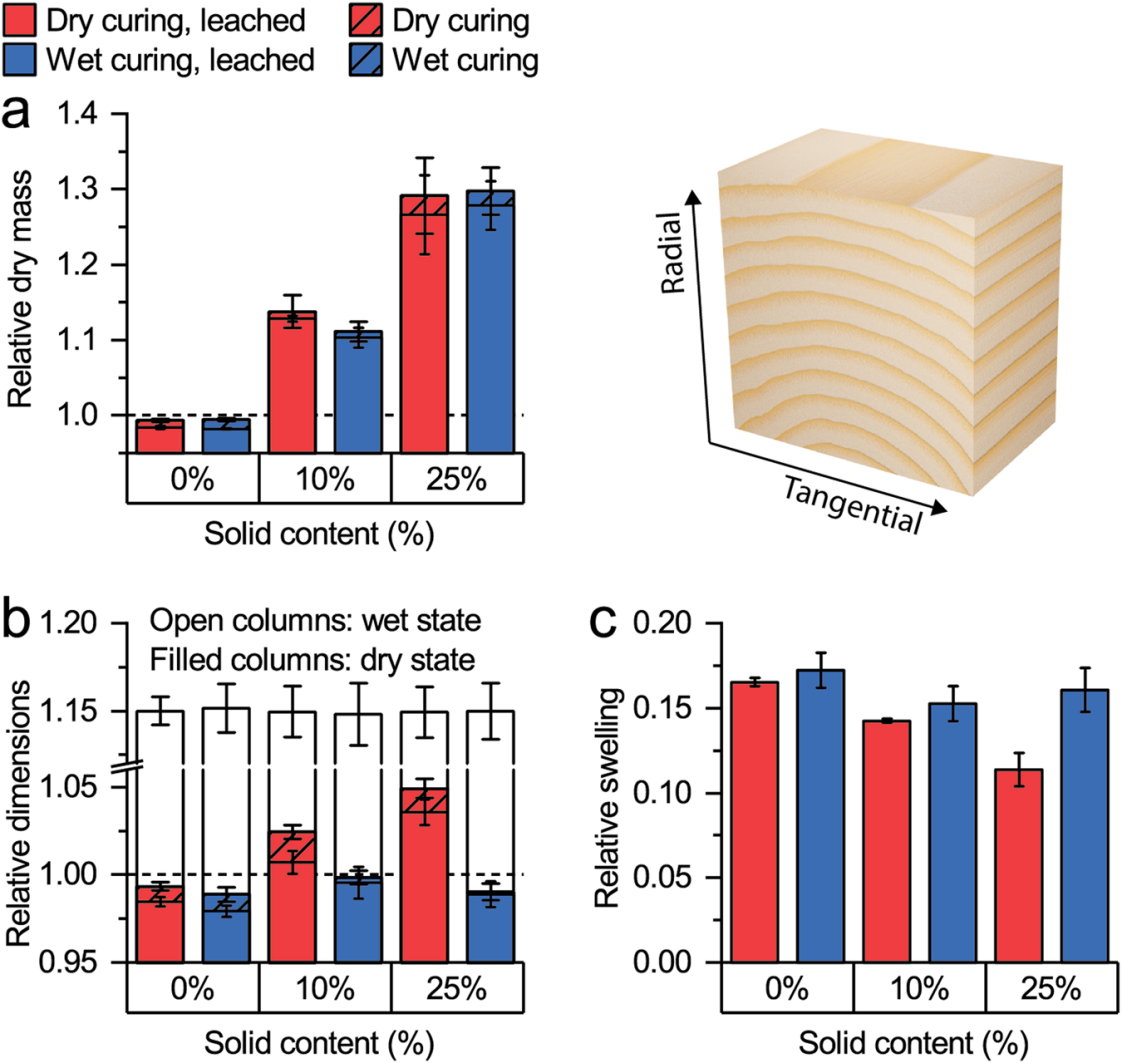
Changes in mass and dimensions measured on wood blocks: Relative dry mass (**a**), relative dimensions (**b**) and relative swelling (**c**) of the dry and wet cured samples. The dashed line highlights y = 1 (no change). Error bars represent the standard deviation of five replicates. Note the break in the y-axis in (**b**).

Our results on macroscopic changes in sample dimensions and mass are in line with previous studies that showed a reduced effectiveness of wet curing conditions to cause a cell wall bulking effect despite a high uptake of MF resin^23^. Changes in sample mass and dimensions were determined on wood blocks and calculated as relative values by relating the dry mass and dimensions after the modification to the initial dry mass and dry dimensions, respectively (Fig. 2). The continuous increase in relative dry mass with increasing solid content of the impregnation solution, even after water leaching, showed the successful fixation of MF resin within the wood samples (Fig. 2a). The curing conditions did not affect the increase in dry sample mass, but mass changes are insensitive to the location of chemical agents within the hierarchical structure of wood. In contrast, an increase in dry dimensions of wood requires the insertion of the modification agent in the cell wall and cannot be realized by simple lumen filling^6,7^. A gradual increase in relative dry dimensions with increasing solid content of the impregnation solution was only determined after dry curing (Fig. 2b), which indicated the diffusion of the MF resin into the cell wall microstructure and a successful cell wall bulking. In contrast, the dry sample dimensions were slightly reduced after wet curing, as shown by relative values below 1. Thus, no cell wall bulking was achieved by wet curing. Wet cured samples were only marginally larger than the reference samples, where leaching of water-soluble extractives caused a small loss in mass and dimensions.

The importance of achieving a cell wall bulking effect was highlighted by the change in relative swelling (Fig. 2c), which describes the dimensional changes of the modified wood samples during drying and rewetting relative to their dry dimensions before modification. A continuous improvement in dimensional stability with increasing solid content was only determined for dry cured samples. Since water-saturated dimensions were not changed compared to the reference values for any of the modified samples (Fig. 2b), the modification did not cause any swelling restraint and the improvement in dimensional stability relied entirely on a cell wall bulking effect, i.e. the blocking of cell wall micropores.

### Scanning electron microscopy

**Figure 3:**
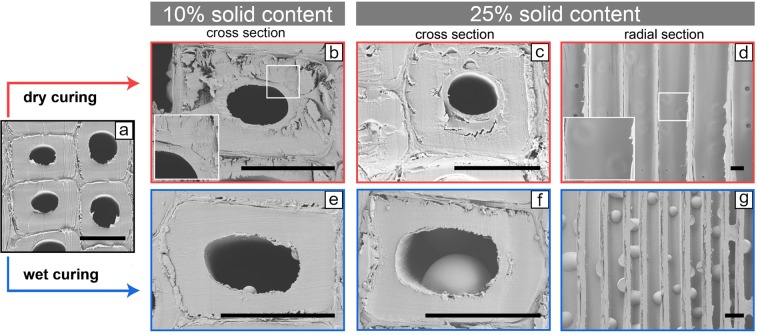
SEM images of Scots pine sapwood that was modified by dry curing (**b**–**d**) and wet curing (**e**–**g**) using solid contents of 10 and 25%. A cross-section of unmodified wood is shown in (**a**). (Scale bars = 20 µm).

The scanning electron microscopy (SEM) images of the modified samples (Fig. 3) showed that many of the cells were filled with MF resin that cured within the cell lumen instead of diffusing into the cell walls. However, lumen filling by MF resin was observed for both heat curing conditions and the SEM observations did not yield quantitative information about differences in the amount of MF resin within the cell lumen. The main difference between wet and dry cured samples was the morphological structure of the lumen fillings. Dry curing resulted in the formation of a circular MF resin layer that covered the lumen surfaces (Fig. 3b,c). On radial sections, such lumen filling was noticed by the lack of structural details of the bordered pits due to coverage by the resin layer (Fig. 3d). In contrast, wet curing resulted in the formation of resin droplets on the lumen surfaces, which were visible on cross-sections (Fig. 3e,f) and radial sections (Fig. 3g). The size of these resin droplets increased with the solid content of the MF resin solution.

MF resin droplets on the lumen surfaces of impregnation modified wood have been observed previously, particularly when drying of the wood samples during curing was reduced by wrapping the samples in plastic bags^24^ or by increasing the relative humidity^26^. Initiating the polycondensation of methylol melamine in aqueous media creates MF microspheres by forming macromolecular aggregates that are poorly solvated in the surrounding medium, which is followed by their growth via coagulation among the water-insoluble polycondensates. This results in MF microspheres with diameters ranging from less than 100 nm to more than 100 µm depending on the reaction time, pH and temperature^27^. Presumably, sufficient amounts of water remained under wet curing conditions to allow the formation of MF microspheres. In contrast, dry curing removed most of the water already at mild temperatures (20–40 °C). Thereby, the MF resin presumably precipitated as a layer on the lumen surface, where it hardened when the temperatures were further increased.

Besides the lumen filling with MF resin, no differences to the reference samples were observed after wet curing. Dry cured samples, however, were very sensitive to the formation of cracks in the secondary cell wall, even in case of an impregnation solution with only 10% solid content. A similar observation was made by Behr *et al*.^26^, who found a higher number of cracks in the cell wall of dry cured beech wood (*Fagus sylvatica* L.) compared to beech wood that was cured at elevated relative humidity.

### Spectroscopic changes of MF resin during heat curing

**Figure 4:**
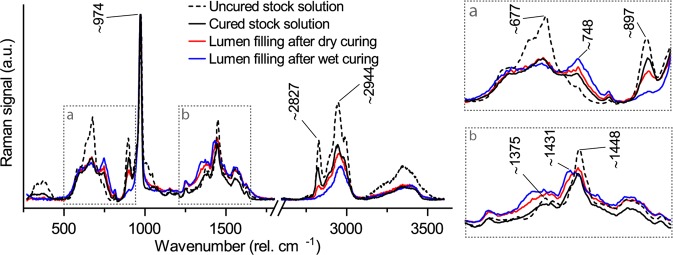
Baseline corrected Raman spectra of the uncured and cured MF resin stock solution as well as Raman spectra of MF resin deposits in the cell lumen of dry and wet cured wood.

Raman spectroscopy enabled the characterization of the change in the MF resin structure induced by heat curing by comparing the spectra of the uncured and cured MF resin stock solution (Fig. 4). The most intense band was found at ca. 974 cm^−1^, which was assigned to the triazine-ring nitrogen radial in-phase vibration of the melamine^28^. This band was insensitive to the resin cure, as reported previously^29,30^. Further bands associated with the triazine ring were found at 677 cm^−1^ (in-plane bending vibration) and at 748 cm^−1^ (out-of-plane bending vibration)^28^. These bands were sensitive to the cure of the MF resin. While the intensity of the band at 748 cm^−1^ increased, the band at 677 cm^−1^ decreased after heat curing, due to the further substitution of the ring during polycondensation^30^. However, the band at 677 cm^−1^ was superimposed on a broad Raman band from ca. 550 to 800 cm^−1^, which was still present even after heat curing the MF resin^30^.

Heat curing also led to a clear decrease in the band intensity at 897 cm^−1^, which was assigned to ether bonds in the resin^29^. One cause for this decrease may have been the cleavage of methyl ether groups in the partially methylated MF resin, which increased the number of methylol groups that contributed to the cross-linking of the MF resin. Furthermore, the transformation of ether-linkages in the partially cured MF resin to methylene bridges by elimination of formaldehyde may have contributed to the loss of ether bonds^14^. The loss in Raman signal for the C-H stretching bands at 2800–3050 cm^−1^ is in line with the cleavage of methyl ether groups as well as the elimination of formaldehyde, because melamine does not contain any C-H units^29^. Methylene bridges are also formed by the reaction of methylol groups with amine groups of melamine^14^. The formation of methylene bridges was observed by the disappearance of the shoulder at around 1000 cm^−1^, as well as by a small decrease at 1448 cm^−1^ along with a shift towards smaller wavenumbers. While methylene bridges have a Raman signal at 1430–1436 cm^−1^, the signal of methylol groups is found at ca. 1448 cm^−1 ^^29,31^.

Spectra collected from the MF resin in the cell lumen of cured wood samples showed the same spectral features as the cured MF resin stock solution (Fig. 4). However, compared with pure MF resin cured using the same temperature sequence, the Raman spectra of the resin deposits in the cell lumen showed a further decrease in the intensities at 677 and 897 cm^−1^, a shift to lower wavenumbers at 1448 cm^−1^ and a further increase at 748 cm^−1^. This was most pronounced for MF resin in wet cured wood and is in line with the suggested catalytic effect of wood on the MF resin cure^16^, which is related to the acidity of wood^17^. While the formation of ether-linkages in MF resin is faster at alkaline pH levels, the reaction rate of methylene bridge formation increases continuously as the pH decreases from 9 to 4^13^. Wet curing may have accelerated the MF resin cure via methylene bridges by the formation of acetic acid from the acetyl groups in hemicelluloses under wet heat conditions^32^, and the enhanced dissociation of carboxylic acids when water is present as a solvent^17^. However, while these spectroscopic data illustrate differences in the MF resin cure, it is unclear how these differences are related to the properties of the resin. Our data do not provide sufficient evidence for differences in the cross-linking density of the cured resins and it is unknown how the MF resin properties are affected by a shift from the formation of ether-linkages to the preferential formation of methylene bridges.

### Spectroscopic changes in modified wood cell walls

**Figure 5:**
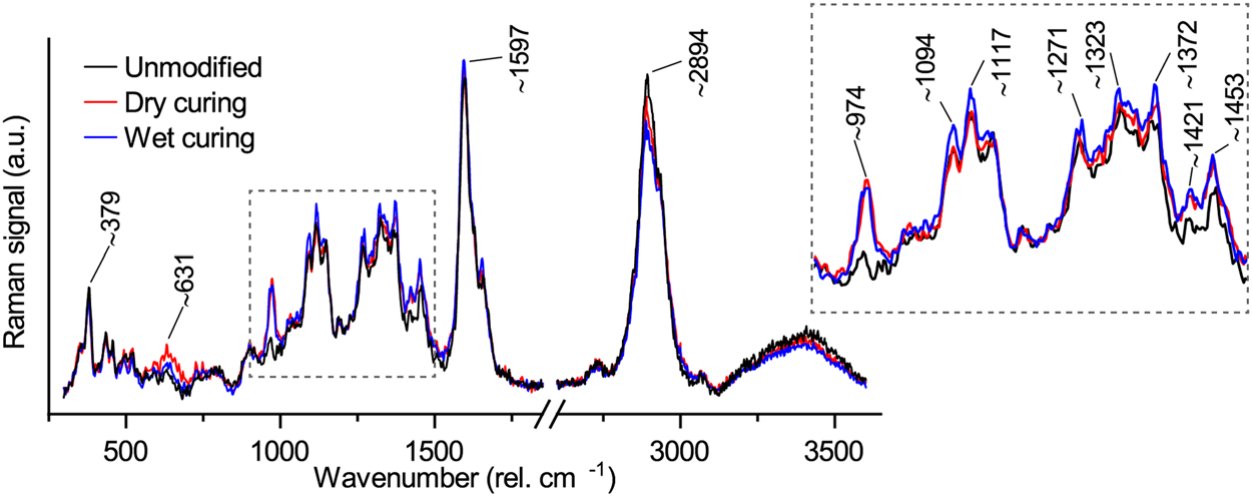
Baseline corrected and normalized average spectra collected from unmodified cell walls and from cell walls that were modified using an impregnation solution with 25% solid content.

The most distinct change in the Raman spectra of modified wood cell walls was the increase in the signal from the triazine ring vibration at ca. 974 cm^−1^, which showed the diffusion of the MF resin into the cell wall (Fig. 5). Furthermore, the modification process led to an increase in the Raman intensities at 1372, 1424 and 1452 cm^−1^, which could have been caused by overlapping of the Raman signals from wood constituents and MF resin. Dry curing also resulted in a small increase at 631 cm^−1^, which could have originated from the broad band structure in MF resin at 550–800 cm^−1^. However, the modification under wet curing conditions also resulted in an increase at 1094 and 1117 cm^−1^, as well as a decrease at ca. 2894 cm^−1^. The latter was also noticed for dry cured cell walls, but was less intense. These spectral changes cannot be explained by Raman signals from the MF resin and depended on the applied curing conditions.

Spectroscopic differences between the applied curing conditions were further determined by principal component analysis (PCA) on an image mosaic of dry and wet cured cells (25% solid content) after preprocessing of the spectra by despiking, baseline correction, normalization and mean centering. Pixels that contained pure MF resin or water were identified by PCA and removed from the image mosaic. The PCA was recalculated using only the remaining mosaic with pixels of the wood cell walls. The first four principal components (PCs) explained ca. 70% of the variation in the data and the following PCs explained only very little variation (Supplementary Fig. S1).

**Figure 6:**
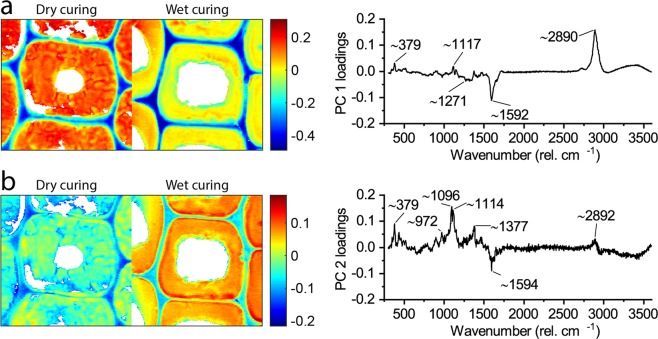
Score images of wood modified using an impregnation solution with 25% solid content (left) and corresponding loading vectors (right) of PC1 (**a**) and PC2 (**b**). The colour scales denote pixel score values.

Score images and corresponding loading vectors of the first two principal components (PCs) are shown in Fig. 6. The loading vector of PC1 had two intense bands: a negative band at ca. 1592 cm^−1^ that originated from aromatics, i.e. lignin, in wood^33^, and a positive band at ca. 2890 cm^−1^. The wavenumber region 2800–3000 cm^−1^ can be assigned to CH/CH_2_ stretching vibrations and generally includes medium lignin bands at 2845–3075 cm^−1^ and strong carbohydrate bands at 2820–2970 cm^−1 ^^33–36^. Consequently, the PC1 score image showed negative scores in the lignin-rich compound middle lamella and cell corners, while positive scores were assigned to the carbohydrate-rich secondary cell wall. A similar separation between compound middle lamella and cell wall was also observed in the PC2 score image. However, the contribution of CH/CH_2_ stretching vibrations was much lower and the PC2 loading vector included stronger contributions from carbohydrate-related bands at ca. 379, 1096, 1114 and 1377 cm^−1 ^^33,37^. Presumably, PC1 separated the signals from lignin and carbohydrates, while PC2 was more sensitive to the crystalline cellulose proportions of the cell wall carbohydrates. This is in line with the increase of the bands at 380 and 1096 cm^−1^ with increasing cellulose crystallinity in lignocelluloses that was shown by Agarwal *et al*.^38^.

PC1 and PC2 also enabled a differentiation between dry and wet cured wood cell walls. The secondary cell walls in wet cured wood had lower PC1 scores, but higher PC2 scores than the secondary cell walls in dry cured wood. Potentially, wet curing removed larger quantities of amorphous carbohydrates (i.e. hemicelluloses), thereby leading to a stronger decrease at the CH/CH_2_ stretching region and lower PC1 scores. Hemicelluloses are removed effectively at alkaline conditions even at temperatures below 100 °C^39^. It is likely that the alkaline MF resin solution caused the hydrolytic cleavage of hemicelluloses and that this proceeded faster when water was present during heat curing under wet conditions. The increase in PC2 scores for the wet cured cell walls may then have been the result of the increase in the proportion of semi-crystalline cellulose. This is in line with Raman measurements of different xylan/cellulose mixtures, which showed an increase at 380 and 1096 cm^−1^ when the xylan (hemicellulose) content in the mixtures decreased^40^.

PC3 and PC4 explained a much lower amount of variation within the data and the respective score images and loading vectors are shown in Supplementary Fig. S2. The PC3 loading vector was dominated by a positive band at ca. 1091 cm^−1^, which is sensitive to the orientation of the cellulose in wood^37^. Therefore, high PC3 scores were found in the S1 layer of the cell wall near the middle lamella, where the cellulose microfibril angle is high. In wet cured wood, high PC3 scores were also observed in the S3 cell wall layer near the lumen. Positive bands in the loading vector of PC4 strongly resembled the spectrum of cured MF resin, in especially the intense band of the triazine-ring vibration at ca. 974 cm^−1^. Accordingly, the PC4 score image marked residual lumen deposits of MF resin near the cell wall interface that were not removed completely from the image mosaic. However, the PCA did not provide evidence for differences in the MF resin cure within the differently modified wood cell walls.

### Distribution of MF resin within wood cell walls

**Figure 7:**
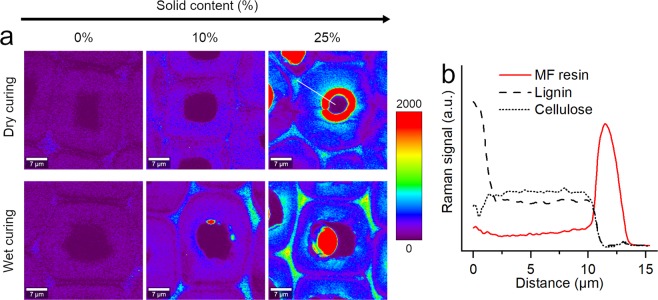
Raman images based on peak integration from 950 to 990 cm^−1^ (**a**). For the dry cured sample at 25% solid content, the distributions of MF resin (950–990 cm^−1^), lignin (1550–1700 cm^−1^) and cellulose (1065–1180 cm^−1^) across the cell wall are shown along the line that is highlighted in the respective Raman image (**b**).

The amount of MF resin in the modified wood was estimated by the peak area at 950–990 cm^−1^, since this peak was not affected by the curing conditions and should correlate with the number of melamine units^29,30^. However, this peak area could not be exclusively assigned to the MF resin, because a small peak in the spectral range 950–990 cm^−1^ was already evident in the reference samples, which originated from cellulose and/or lignin in native wood (Fig. 5b)^33,41^. Consequently, it was not feasible to accurately quantify the concentration of MF resin within the cell wall. Nonetheless, a gradual increase in the peak area at 950–990 cm^−1^ with increasing solid content of the impregnation solution was found for latewood cell walls (Fig. 7a). This was a clear indication of the diffusion of the MF resin into the cell wall that was driven by the concentration gradient between cell lumen and cell wall. Further evidence was provided by the continuous increases in the peak area ratios of MF resin (950–990 cm^−1^) to lignin (1550–1700 cm^−1^) and to cellulose (1080–1175 cm^−1^), which were based on average spectra that were collected from the S_2_ cell wall layers of a number of latewood cells (Supplementary Fig. S3).

A continuous increase in the peak area at 950–990 cm^−1^ (Fig. 7a) and in the peak area ratios (Supplementary Fig. S3) with increasing solid contents was also found after wet curing. The peak area ratios were only slightly lower after wet curing compared to dry curing. This showed that MF resin had also diffused into the cell walls under wet curing conditions despite no increase in the dry sample dimensions. A possible explanation of this contradiction is the enhanced alkaline hydrolysis of the cell wall during wet curing, which was also indicated by the PCA. Presumably, MF resin had mostly filled cell wall pores that were created by the removal of cell wall compounds under wet curing conditions and, thus, the dry dimensions did not increase. In contrast, the removal of cell wall constituents was less intense during dry curing and, consequently, the MF resin caused a permanent swelling of the cell wall (cell wall bulking). Additional treatments of wood with an alkaline carbonate-bicarbonate buffer without MF resin confirmed the effects of alkaline hydrolysis under the modification conditions applied. Using the alkaline buffer solution instead of water and applying wet instead of dry curing conditions led to a further decrease in dry mass and dry dimensions (Supplementary Fig. S4). The hydrolytic cleavage of covalent bonds during these additional treatments was shown by the loss in absorbance in the mid-infrared spectra at ca. 1733 (C=O stretching in COOH) and 1230 cm^−1^ (C-O-C asymmetric stretching vibration), which followed the same trend as the loss in wood mass (Supplementary Fig. S5). This was presumably caused by the hydrolytic cleavage of acetyl groups from the hemicelluloses.

The results also suggested that the impact of the curing conditions on the cell wall diffusion was smaller than suggested previously^23^. Presumably, high quantities of MF resin had already diffused into the cell wall during the impregnation and the exposure to mild temperatures (20 °C) at the beginning of the curing stage. Furthermore, dry curing not only removed water from the lumen to enhance cell wall diffusion, but presumably also removed water from the cell wall at an early curing stage, which reduced the water-swollen porosity and the diffusivity of MF resin into the cell wall^22^.

Nonetheless, some differences between dry and wet curing conditions in the diffusion of the MF resin across the cell walls were found. In line with previous studies^19,24,25,42^, a comparably high concentration of MF resin was often observed in the cell corners, in particular for wet cured samples (Fig. 7a). In addition to the direct transport from the lumen into the secondary cell wall, MF resin and other solutes may have diffused from the lumen over the pit membranes through the interconnected middle lamella and cell corners^19,43–45^. Wet curing may have supported the cure of the MF resin to macromolecules in the cell corners before its diffusion into the secondary cell wall. The Raman images also suggested a gradient in the amount of MF resin across the secondary cell wall, with higher amounts of MF resin in the inner cell wall layers near the cell lumen of wood treated with a 25% solid content (Fig. 7a). When following the change in the peak areas at 950–990 cm^−1^ (mainly MF resin), 1065–1180 cm^−1^ (cellulose) and 1550–1700 cm^−1^ (lignin) across a dry cured cell wall, the amount of MF resin decreased continuously from the cell wall-lumen interface towards the S_1_ layer of the cell wall and increased again towards the cell corner (Fig. 7b). A higher amount of MF resin near the lumen interface would suggest a higher cell wall bulking in these cell wall regions. This may have contributed effectively to the increase in sample dimensions, especially since this enhanced MF resin uptake of the inner cell wall layers would cover a larger proportion of the thin earlywood cell walls^9^. However, increased amounts of MF resin near the lumen interface were also found in wet cured cell walls and also in some distance to lumen deposits. Therefore, it remains uncertain if the curing conditions or the shape of the lumen deposits had any impact on the enhanced uptake of MF resin within the inner cell wall layers.

## Conclusions

Our results showed that curing of MF resin within the wood promoted the cross-linking of the resin via methylene bridges, especially under wet curing conditions. The curing conditions also affected the morphological structure of the deposits of MF resin in the cell lumen, resulting either in resin droplets under wet conditions or in resin films on the cell wall surface under dry conditions. Furthermore, our results showed that macroscopic changes in sample dimensions were not only the result of the diffusion of MF resin into the cell wall to occupy cell wall pores. The removal of cell wall constituents by the exposure to heat and an alkaline impregnation solution counterbalanced the cell wall bulking effect caused by the resin. Wet curing conditions further facilitated this alkaline hydrolysis within the cell wall. Therefore, no increase in the sample dimensions was measured after wet curing, despite a diffusion of MF resin into the cell wall.

## Material and Methods

### Sample preparation

Scots pine (*Pinus sylvestris* L.) sapwood samples with dimensions of 25 × 25 × 10 mm^3^ (radial × tangential × longitudinal) were prepared from commercially kiln-dried boards. The samples used for the experiments had an average dry density of 0.5 (± 0.06) g cm^−3^ and no visible defects. All samples were conditioned at 20 °C and 65% RH prior to any treatment.

### Impregnation modification

The wood samples (n = 5) were vacuum-impregnated at 100 mbar for 1 h with an aqueous solution of low molecular weight MF resin (Madurit MW 840; INEOS Melamines GmbH, Germany). Methylol groups of the MF resin were partly methylated. The MF resin was supplied as an aqueous stock solution with a pH of 10.1. Non-volatile matter of the stock solution was determined as percentage residue after drying at 103 °C for 24 h. Based on the result, solutions with solid contents of 10 and 25% were prepared by dilution with deionized water. Pure deionized water was used as a reference. After impregnation, the samples were left in the solution at ambient pressure for 1 h, before they were removed from the solution and exposed to the following temperature sequence: 20, 40, 60, 80, 40 and 103 °C, with each step being held for 24 h. For wet curing, the specimens were wrapped in aluminium foil for the first four temperature steps. For dry curing, the samples remained unwrapped. Five samples per sample group (solid content and curing condition) were treated. After the modification, all samples were vacuum-impregnated with deionized water and leached for one week with daily water changes.

Using the same procedure, wood samples (n = 5) were treated with a 0.2 M carbonate-bicarbonate buffer (pH = 10) instead of the MF resin solution. Again, controls were treated with deionized water. Furthermore, cured MF resin was prepared by heating ca. 5 g of the undiluted stock solution at the same temperature sequence that was applied to cure the impregnated wood samples.

### Determination of mass and dimensional changes

Changes in mass and dimensions (radial × tangential) were determined in oven-dry state directly after curing, in water-saturated state at the end of the water leaching protocol and after re-drying at 103 °C for 24 h. The dry mass and dimensions are given as relative values by relating each mass and dimension to the original dry mass and dry dimension of the sample before the modification. Relative swelling was calculated by subtracting the relative dry dimensions from the relative wet dimensions.

### Infrared spectroscopy

Fourier transform infrared (FT-IR) spectra of wood treated with water or the carbonate-bicarbonate buffer were measured using a FT-IR spectrometer (Spectrum Two, PerkinElmer, USA) equipped with an ATR unit and a diamond crystal. Spectra were acquired within the wavenumber region 4000–750 cm^−1^ at a resolution of 4 cm^−1^ and 8 accumulations. The spectra were baseline corrected, and normalized by the area below each spectrum.

### Scanning electron microscopy (SEM)

Smooth surfaces of the wood samples were created using water soaked samples and a rotary microtome. Samples were dried and coated with 4 nm of gold. Images were taken with a scanning electron microscope (Zeiss, Sigma VP, Germany) using a beam acceleration voltage of 2 kV and a detector for secondary electrons.

### Confocal Raman microscopy

Raman spectra and images were acquired with a WITec alpha 300 RA Raman microscope (WITec, Ulm, Germany) equipped with a 532 nm frequency doubled Nd:YAG laser and a DU970-BV EMCCD camera behind a 600 lines/mm grating. Single spectra of pure MF resin were acquired using a 20× air objective (numerical aperture (NA) = 0.4), an integration time of 0.5 s and 10 accumulations. To measure wood cells, cross-sections with 20 µm thickness were cut on a rotary microtome, placed on objective slides with a drop of deionized water, covered with a glass coverslip (0.17 mm thickness) and edge sealed with nail polish. From the sections, Raman images were acquired with a 100× immersion oil objective (NA = 1.25, coverslip correction 0.17 mm). The size of each image was 40 × 40 μm^2^, with 175 lines per image and 175 points per line. An integration time of 0.3 s was applied. The measurements were performed at least 3 months after the heat cure of the samples and were limited to latewood cells, but images were taken at different locations within each sample.

Cosmic ray removal and baseline correction was done using the WITec Suite 5.1 software. Average spectra of wood cell walls and MF resin deposits within cell lumens were collected from Raman images. They were baseline corrected by fitting a fifth order polynomial over selected wavenumber areas. Wood cell wall spectra were normalized by calculating the sum of the squared intensity values of each spectrum and using the squared root of this sum as the normalisation constant. Peak areas were calculated without any prior baseline correction or normalization by integration over specific wavelength ranges, with the background being set to zero at four wavenumbers below and above the respective range.

### Multivariate image analysis

Selected Raman images were combined into an image mosaic for PCA, as described previously^46^. The image mosaic was unfolded into a two-dimensional array with individual pixels as row objects and wavenumbers as the corresponding columns. Wavelengths outside the range 300–3600 cm^−1^ were excluded. Cosmic rays and obvious outliers were removed with an image filter that replaced an outlier with the median spectrum of a moving window. Baseline correction was performed by fitting a 3^rd^ order polynomial over the selected wavenumber area and normalization was done by calculating the sum of the squared intensity values of the spectrum and using the squared root of this sum as the normalisation constant. PCA was done after mean centering of the preprocessed spectra. The first PC was used to remove pixels that contained mostly water or pure MF resin based on a set score value threshold (see Supplementary Fig. S6). Using only the remaining data set, mean centering was performed again and the PCA was recalculated. The scores were refolded back to the image dimension to allow visualization of the results. Data analysis was performed through a combination of in-house MATLAB (MathWorks, Inc.) scripts and commercial functions from the PLS Toolbox (Eigenvector Research, Inc.).

## Supplementary information

Supplementary information.

## Data Availability

The datasets generated and/or analysed during the current study are available from the corresponding author on reasonable request.
