# Supplementary material for: Distribution and curing reactions of melamine formaldehyde resin in cells of impregnation-modified wood
Source: Sci Rep. 2020 Feb 25;10:3366. doi: 10.1038/s41598-020-60418-3 (PMC7042241; doi:10.1038/s41598-020-60418-3)
Supplement: Supplementary file 1 — Supplementary information. [file 41598_2020_60418_MOESM1_ESM.pdf]

Supplementary material

Distribution and curing reactions of melamine formaldehyde resin in cells of impregnation-modified wood

Michael Altgen[1]*, Muhammad Awais[1], Daniela Altgen[1], André Klüppel[2], Mikko Mäkelä[1,3], Lauri Rautkari[1]

*corresponding author, michael.altgen@aalto.fi

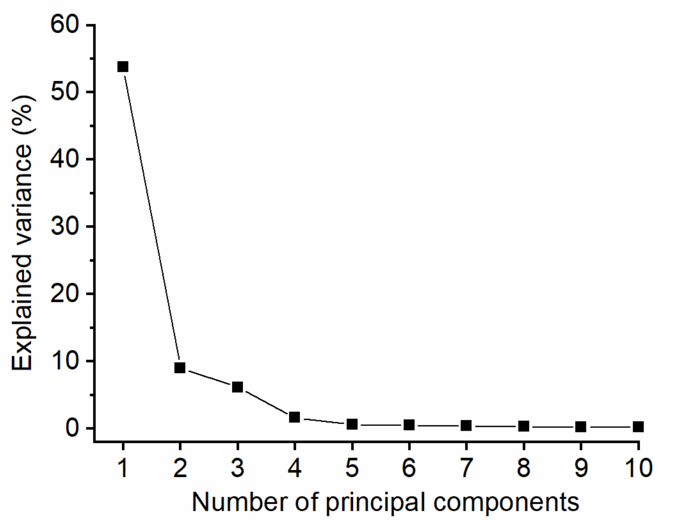

Fig. S. 1: Scree plot of variance. The first four principal components explained a total of ca. 70 % of the variance within the data.

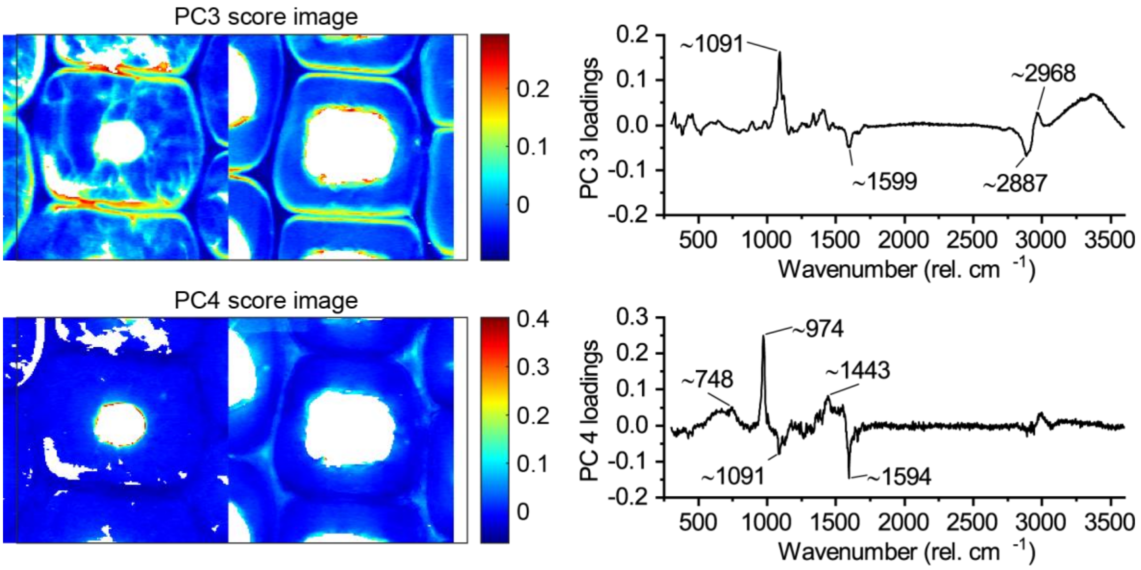

Fig. S. 2: Score images and corresponding loading vectors of the first three principal components based on an image mosaic of dry and wet cured latewood cells (25 % solid content). The color scales denote pixel score values. Pixels containing pure MF resin and water were removed.

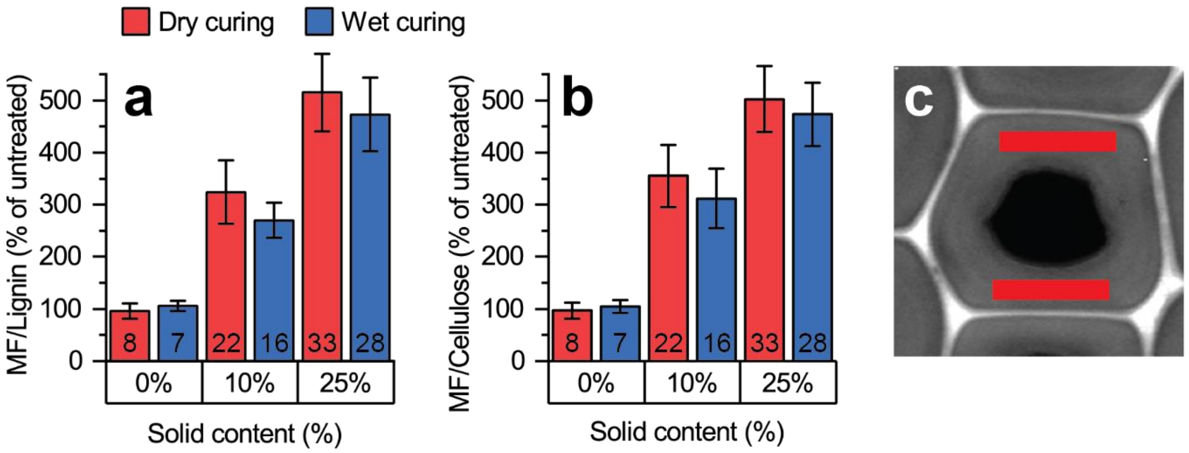

Fig. S. 3: Peak area ratios of MF resin (950-990 cm^{-1}) to lignin (1550-1700 cm^{-1}; a) and to cellulose (1065-1180 cm^{-1}; b). The results were normalized to the peak area ratios of untreated Scots pine sapwood (=100%). An example for the areas within the wood cell from which the average spectra were taken is shown in (c). The number of average spectra is shown at the bottom of each column. Error bars show the standard deviation.

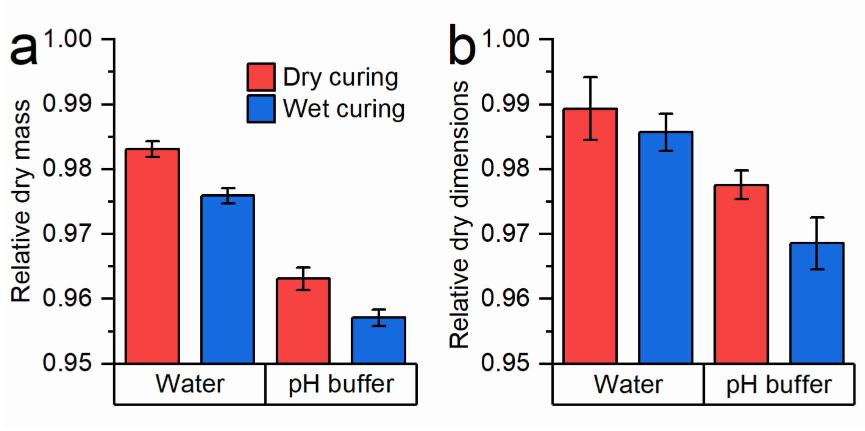

Fig. S. 4: Relative dry mass (a) and relative dry dimensions (b) in dependence of the impregnation solution (deionized water or alkaline pH buffer solution) and after dry or wet curing. (N=5; ±standard deviation).

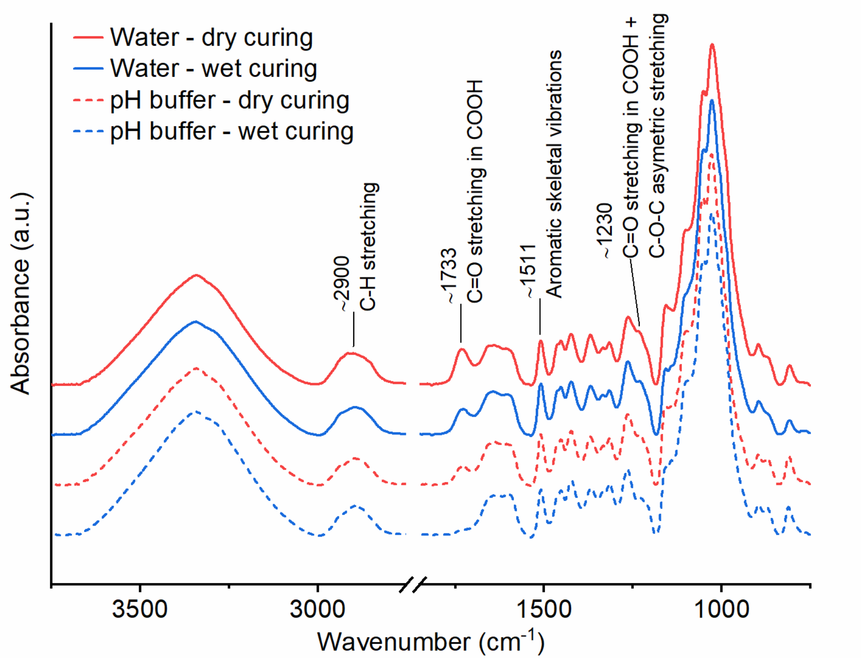

Fig. S. 5: FT-IR spectra of wood after treatments with deionized water or an alkaline carbonate-bicarbonate buffer solution under dry or wet curing conditions.

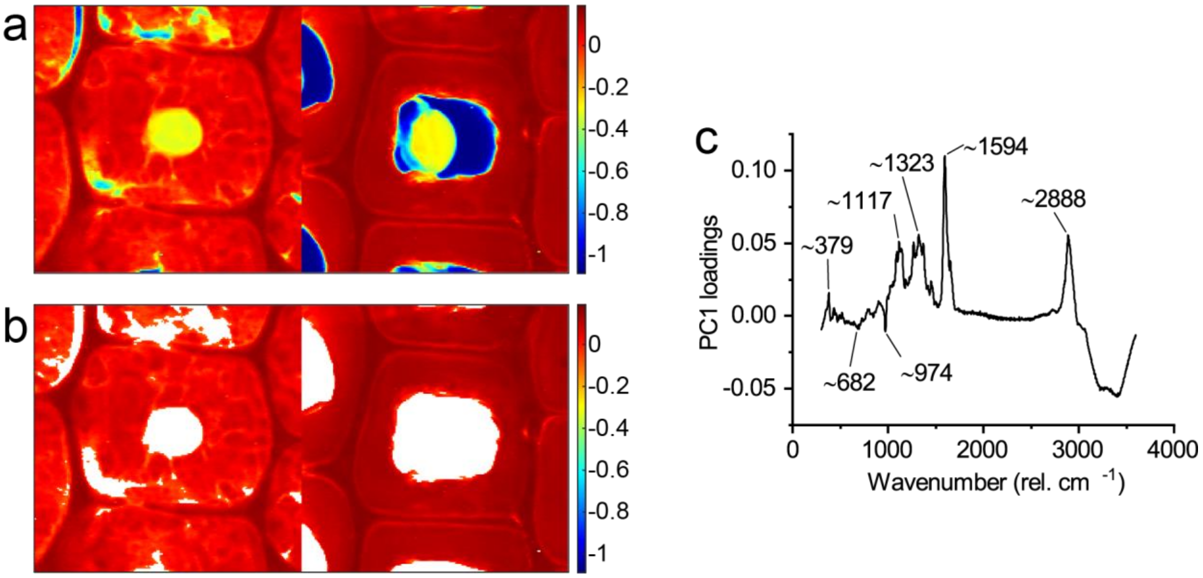

Fig. S. 6: PC1 score image before (a) and after (b) removing pixels with a score value lower than -0.1. The corresponding loading vector is shown in (c).
